# Heptane-1,7-diaminium dinitrate

**DOI:** 10.1107/S1600536811042917

**Published:** 2011-10-22

**Authors:** Charmaine Arderne

**Affiliations:** a University of Johannesburg, Department of Chemistry, PO Box 524, Auckland Park, Johannesburg 2006, South Africa

## Abstract

In the title molecular salt, C_7_H_20_N_2_^2+^·2NO_3_^−^, the crystal structure exhibits an unusual back-to-back paired double-stacked packing arrangement culminating in an overall double zigzag pattern of the dications. The nitrate anions form a ring around one pair of double-stacked dications. An intricate three-dimensional N—H⋯O and N—H⋯(O,O) hydrogen-bonding network exists in the crystal structure.

## Related literature

For related structural studies of *n*-alkyl-diammonium nitrate salts, see: van Blerk & Kruger (2009 ▶). For the Cambridge Structural Database, see: Allen (2002 ▶).
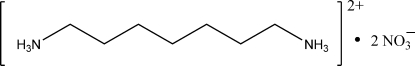


## Experimental

### 

#### Crystal data

C_7_H_20_N_2_^2+^·2NO_3_^−^
*M* *_r_* = 256.27
Monoclinic, 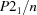

*a* = 5.3236 (1) Å
*b* = 16.8340 (4) Å
*c* = 14.9845 (3) Å
β = 96.500 (1)°
*V* = 1334.24 (5) Å^3^
*Z* = 4
Mo *K*α radiation
μ = 0.11 mm^−1^
*T* = 295 K
0.44 × 0.35 × 0.32 mm

#### Data collection

Bruker SMART CCD diffractometer
Absorption correction: multi-scan (AX-SCALE; Bruker, 2008 ▶) *T*_min_ = 0.953, *T*_max_ = 0.966
16750 measured reflections
2343 independent reflections
1828 reflections with *I* > 2σ(*I*)
*R*_int_ = 0.032

#### Refinement

*R*[*F* ^2^ > 2σ(*F* ^2^)] = 0.042
*wR*(*F* ^2^) = 0.124
*S* = 1.06
2343 reflections
157 parameters
H-atom parameters constrained
Δρ_max_ = 0.29 e Å^−3^
Δρ_min_ = −0.18 e Å^−3^

### 

Data collection: *SMART-NT* (Bruker, 1999 ▶); cell refinement: *SAINT* (Bruker, 2008 ▶); data reduction: *SAINT*; program(s) used to solve structure: *SHELXS97* (Sheldrick, 2008 ▶); program(s) used to refine structure: *SHELXL97* (Sheldrick, 2008 ▶); molecular graphics: *OLEX2* (Dolomanov *et al.*, 2009 ▶) and *Mercury* (Macrae *et al.*, 2008 ▶); software used to prepare material for publication: *publCIF* (Westrip, 2010 ▶) and *PLATON* (Spek, 2009 ▶).

## Supplementary Material

Crystal structure: contains datablock(s) I, global. DOI: 10.1107/S1600536811042917/ez2262sup1.cif

Structure factors: contains datablock(s) I. DOI: 10.1107/S1600536811042917/ez2262Isup2.hkl

Supplementary material file. DOI: 10.1107/S1600536811042917/ez2262Isup3.mol

Supplementary material file. DOI: 10.1107/S1600536811042917/ez2262Isup4.cml

Additional supplementary materials:  crystallographic information; 3D view; checkCIF report

## Figures and Tables

**Table 1:** Hydrogen-bond geometry (Å, °)

*D*—H⋯*A*	*D*—H	H⋯*A*	*D*⋯*A*	*D*—H⋯*A*
N1—H1*C*⋯O4^i^	0.89	2.16	2.956 (2)	149
N1—H1*C*⋯O6^ii^	0.89	2.62	3.129 (2)	118
N1—H1*D*⋯O1^ii^	0.89	2.09	2.951 (2)	161
N1—H1*D*⋯O3^ii^	0.89	2.40	3.035 (2)	129
N1—H1*E*⋯O1^i^	0.89	2.04	2.871 (2)	155
N1—H1*E*⋯O2^i^	0.89	2.43	3.208 (3)	147
N2—H2*C*⋯O2^iii^	0.89	2.26	3.142 (2)	172
N2—H2*C*⋯O3^iii^	0.89	2.26	2.911 (2)	130
N2—H2*D*⋯O4^iv^	0.89	2.07	2.901 (2)	155
N2—H2*E*⋯O4^v^	0.89	2.44	3.0064 (19)	122
N2—H2*E*⋯O6^v^	0.89	2.08	2.967 (2)	171

Symmetry codes: (i) 
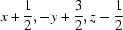
; (ii) 
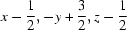
; (iii) 
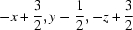
; (iv) 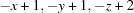; (v) 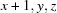.

**Fig. 1.**
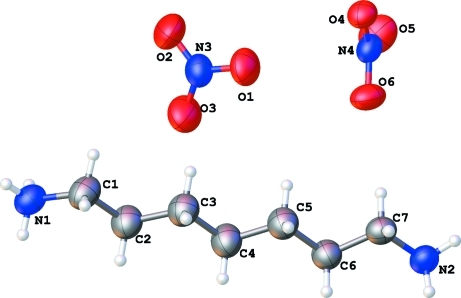
Molecular structure of the title compound, with atomic numbering scheme and displacement ellipsoids drawn at the 50% probability level.

**Fig. 2.**
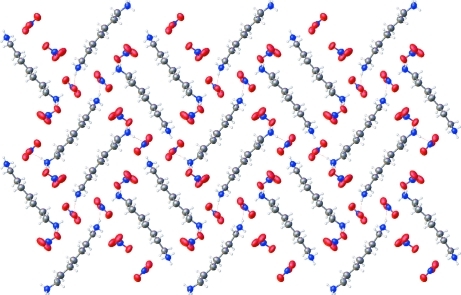
Packing arrangement of the title compound viewed down the a axis. Selected hydrogen bonds are indicated by red dashed lines.

**Fig. 3.**
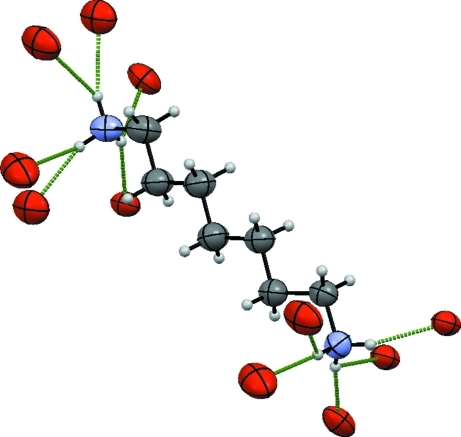
Close-up view of the title compound clearly showing selected hydrogen-bonding interactions. Hydrogen bonds are indicated by green dashed lines.

## supplementary crystallographic information

### Comment

The crystal structure of the title compound (I) adds to our current ongoing investigations of long-chained diammonium inorganic mineral acid salts (van Blerk & Kruger, 2009). Colourless crystals of heptane-1,7-diammonium dinitrate were obtained and analyzed by single-crystal X-ray diffraction techniques. This material forms part of our structural chemistry study of the inorganic mineral acid salts of the *n*-alkyldiamines. A search of the Cambridge Structural Database (Version 5.32, Allen, 2002) revealed that this compound had not previously been determined.

The asymmetric unit of compound (I) contains one diammonium dication and two nitrate anions with all atoms occupying general positions. The hydrocarbon chain is also fully extended with very slight deviations from planarity chain as is evident from the torsion angles along the hydrocarbon chain (tabulated in Table 1). The molecular structure of (I) is shown in Fig. 1.

Fig. 2 illustrates the packing arrangement of the title compound (I) viewed down the *a* axis. The diammonium cations pack back-to-back, in pairs in a double zig-zag pattern. Each dication pair is completely surrounded by a ring of nitrate anions. An extensive three-dimensional hydrogen-bonding network is also formed of N—H···O hydrogen bonds.

A close-up view of selected hydrogen bonding interactions can be viewed in Fig. 3. The three-dimensional hydrogen bonding network is built and linked through hydrogen bonding interactions between the ammonium groups of the dication and the nitrate anions. Clear evidence of bifurcated hydrogen bonding interactions can also be seen in this illustration. The hydrogen bond distances and angles for the title compound (I) can be found in Table 2.

### Experimental

Compound (I) was prepared by adding heptane-1,7-diamine (0.50 g, 3.84 mmol) to 55% nitric acid (2 ml, 42.5 mmol, Merck) in a sample vial. The mixture was then refluxed at 363 K for 2 h. The solution was cooled at 2 K h^-1^ to room temperature. Colourless crystals of heptane-1,7-diammonium dinitrate were collected and a suitable single-crystal was selected for the X-ray diffraction study.

### Refinement

H atoms were geometrically positioned and refined in the riding-model approximation, with C—H = 0.97 Å, N—H = 0.89 Å, and *U*_iso_(H) = 1.2*U*_eq_(C) or 1.5*U*_eq_(N). For (I), the highest peak in the final difference map is 1.05 Å from O3 and the deepest hole is 0.87 Å from O3.

### Crystal data

**Table:**

C_7_H_20_N_2_^2+^·2NO_3_^−^	*F*(000) = 552
*M**_r_* = 256.27	*D*_x_ = 1.276 Mg m^−^^3^
Monoclinic, *P*2_1_/*n*	Mo *K*α radiation, λ = 0.71073 Å
Hall symbol: -P 2yn	Cell parameters from 8072 reflections
*a* = 5.3236 (1) Å	θ = 2.4–24.5°
*b* = 16.8340 (4) Å	µ = 0.11 mm^−^^1^
*c* = 14.9845 (3) Å	*T* = 295 K
β = 96.500 (1)°	Block, colourless
*V* = 1334.24 (5) Å^3^	0.44 × 0.35 × 0.32 mm
*Z* = 4	

### Data collection

**Table:**

Bruker SMART CCD diffractometer	2343 independent reflections
Radiation source: fine-focus sealed tube	1828 reflections with *I* > 2σ(*I*)
graphite	*R*_int_ = 0.032
φ and ω scans	θ_max_ = 25.0°, θ_min_ = 1.8°
Absorption correction: multi-scan (AX-SCALE; Bruker, 2008)	*h* = −6→6
*T*_min_ = 0.953, *T*_max_ = 0.966	*k* = −19→19
16750 measured reflections	*l* = −17→17

### Refinement

**Table:**

Refinement on *F*^2^	Secondary atom site location: difference Fourier map
Least-squares matrix: full	Hydrogen site location: inferred from neighbouring sites
*R*[*F*^2^ > 2σ(*F*^2^)] = 0.042	H-atom parameters constrained
*wR*(*F*^2^) = 0.124	*w* = 1/[σ^2^(*F*_o_^2^) + (0.0558*P*)^2^ + 0.3767*P*] where *P* = (*F*_o_^2^ + 2*F*_c_^2^)/3
*S* = 1.06	(Δ/σ)_max_ < 0.001
2343 reflections	Δρ_max_ = 0.29 e Å^−^^3^
157 parameters	Δρ_min_ = −0.18 e Å^−^^3^
0 restraints	Extinction correction: *SHELXL97* (Sheldrick, 2008), Fc^*^=kFc[1+0.001xFc^2^λ^3^/sin(2θ)]^-1/4^
Primary atom site location: structure-invariant direct methods	Extinction coefficient: 0.027 (3)

### Special details

**Table:**

Geometry. All e.s.d.'s (except the e.s.d. in the dihedral angle between two l.s. planes) are estimated using the full covariance matrix. The cell e.s.d.'s are taken into account individually in the estimation of e.s.d.'s in distances, angles and torsion angles; correlations between e.s.d.'s in cell parameters are only used when they are defined by crystal symmetry. An approximate (isotropic) treatment of cell e.s.d.'s is used for estimating e.s.d.'s involving l.s. planes.
Refinement. Refinement of *F*^2^ against ALL reflections. The weighted *R*-factor *wR* and goodness of fit *S* are based on *F*^2^, conventional *R*-factors *R* are based on *F*, with *F* set to zero for negative *F*^2^. The threshold expression of *F*^2^ > σ(*F*^2^) is used only for calculating *R*-factors(gt) *etc*. and is not relevant to the choice of reflections for refinement. *R*-factors based on *F*^2^ are statistically about twice as large as those based on *F*, and *R*-factors based on ALL data will be even larger.

### Fractional atomic coordinates and isotropic or equivalent isotropic displacement parameters (Å^2^)

**Table:**

	*x*	*y*	*z*	*U*_iso_*/*U*_eq_	
C1	0.2521 (4)	0.71038 (12)	0.43729 (16)	0.0700 (6)	
H1A	0.1900	0.6662	0.3994	0.084*	
H1B	0.1282	0.7213	0.4784	0.084*	
C2	0.4951 (4)	0.68708 (14)	0.48992 (15)	0.0703 (6)	
H2A	0.5671	0.7331	0.5223	0.084*	
H2B	0.6127	0.6703	0.4487	0.084*	
C3	0.4660 (4)	0.62061 (13)	0.55650 (14)	0.0683 (6)	
H3A	0.3515	0.6380	0.5986	0.082*	
H3B	0.3899	0.5751	0.5243	0.082*	
C4	0.7127 (4)	0.59522 (14)	0.60854 (15)	0.0724 (6)	
H4A	0.8182	0.5710	0.5673	0.087*	
H4B	0.8000	0.6420	0.6339	0.087*	
C5	0.6812 (4)	0.53735 (13)	0.68327 (14)	0.0670 (5)	
H5A	0.5906	0.4911	0.6580	0.080*	
H5B	0.5781	0.5620	0.7249	0.080*	
C6	0.9287 (4)	0.50997 (13)	0.73523 (13)	0.0617 (5)	
H6A	1.0223	0.4783	0.6962	0.074*	
H6B	1.0306	0.5560	0.7543	0.074*	
C7	0.8826 (3)	0.46172 (11)	0.81595 (13)	0.0571 (5)	
H7A	0.7854	0.4931	0.8541	0.068*	
H7B	0.7831	0.4153	0.7965	0.068*	
N1	0.2774 (3)	0.78115 (9)	0.38028 (11)	0.0612 (4)	
H1C	0.3176	0.8232	0.4150	0.092*	
H1D	0.1315	0.7901	0.3466	0.092*	
H1E	0.3983	0.7727	0.3449	0.092*	
N2	1.1202 (3)	0.43572 (9)	0.86872 (10)	0.0563 (4)	
H2C	1.1956	0.3994	0.8378	0.084*	
H2D	1.0855	0.4148	0.9205	0.084*	
H2E	1.2224	0.4773	0.8796	0.084*	
N3	0.2584 (3)	0.77064 (11)	0.75577 (11)	0.0643 (5)	
N4	0.3365 (3)	0.60428 (9)	0.97456 (10)	0.0524 (4)	
O1	0.2441 (3)	0.70920 (9)	0.80187 (11)	0.0828 (5)	
O2	0.0960 (4)	0.82199 (13)	0.75256 (13)	0.1094 (7)	
O3	0.4470 (3)	0.78063 (11)	0.71661 (13)	0.0983 (6)	
O4	0.1024 (2)	0.59148 (8)	0.96594 (8)	0.0579 (4)	
O5	0.4312 (3)	0.65102 (10)	1.03157 (11)	0.0859 (5)	
O6	0.4699 (3)	0.56836 (8)	0.92489 (11)	0.0731 (4)	

### Atomic displacement parameters (Å^2^)

**Table:**

	*U*^11^	*U*^22^	*U*^33^	*U*^12^	*U*^13^	*U*^23^
C1	0.0538 (11)	0.0625 (12)	0.0923 (15)	−0.0014 (9)	0.0016 (10)	0.0079 (11)
C2	0.0556 (11)	0.0777 (14)	0.0755 (13)	−0.0033 (10)	−0.0012 (10)	0.0081 (11)
C3	0.0584 (12)	0.0721 (13)	0.0727 (13)	−0.0007 (10)	−0.0003 (10)	0.0041 (11)
C4	0.0593 (12)	0.0834 (15)	0.0729 (13)	−0.0023 (11)	0.0004 (10)	0.0120 (11)
C5	0.0561 (11)	0.0755 (13)	0.0683 (12)	−0.0021 (10)	0.0020 (9)	0.0050 (10)
C6	0.0528 (11)	0.0696 (12)	0.0624 (11)	−0.0009 (9)	0.0045 (9)	0.0033 (10)
C7	0.0504 (10)	0.0580 (11)	0.0631 (11)	0.0000 (8)	0.0078 (8)	−0.0033 (9)
N1	0.0513 (9)	0.0562 (9)	0.0743 (11)	0.0003 (7)	0.0001 (8)	−0.0044 (8)
N2	0.0582 (9)	0.0552 (9)	0.0548 (9)	0.0062 (7)	0.0033 (7)	−0.0041 (7)
N3	0.0607 (10)	0.0703 (11)	0.0601 (10)	−0.0032 (9)	−0.0014 (8)	0.0114 (8)
N4	0.0455 (9)	0.0497 (9)	0.0609 (9)	0.0030 (7)	0.0016 (7)	0.0092 (7)
O1	0.0805 (11)	0.0728 (10)	0.0962 (11)	−0.0081 (8)	0.0145 (8)	0.0310 (9)
O2	0.0942 (13)	0.1239 (15)	0.1122 (14)	0.0452 (12)	0.0216 (11)	0.0440 (12)
O3	0.0797 (11)	0.1003 (13)	0.1192 (14)	−0.0011 (9)	0.0293 (10)	0.0406 (11)
O4	0.0425 (7)	0.0639 (8)	0.0674 (8)	0.0010 (6)	0.0061 (6)	0.0046 (6)
O5	0.0768 (11)	0.0803 (11)	0.0948 (11)	−0.0076 (8)	−0.0155 (9)	−0.0181 (9)
O6	0.0574 (8)	0.0696 (9)	0.0967 (11)	0.0094 (7)	0.0286 (8)	0.0016 (8)

### Geometric parameters (Å, °)

**Table:**

C1—N1	1.481 (3)	C6—H6A	0.9700
C1—C2	1.489 (3)	C6—H6B	0.9700
C1—H1A	0.9700	C7—N2	1.480 (2)
C1—H1B	0.9700	C7—H7A	0.9700
C2—C3	1.519 (3)	C7—H7B	0.9700
C2—H2A	0.9700	N1—H1C	0.8900
C2—H2B	0.9700	N1—H1D	0.8900
C3—C4	1.511 (3)	N1—H1E	0.8900
C3—H3A	0.9700	N2—H2C	0.8900
C3—H3B	0.9700	N2—H2D	0.8900
C4—C5	1.508 (3)	N2—H2E	0.8900
C4—H4A	0.9700	N3—O2	1.220 (2)
C4—H4B	0.9700	N3—O3	1.230 (2)
C5—C6	1.524 (3)	N3—O1	1.251 (2)
C5—H5A	0.9700	N4—O5	1.227 (2)
C5—H5B	0.9700	N4—O6	1.2424 (19)
C6—C7	1.500 (3)	N4—O4	1.2570 (18)
			
N1—C1—C2	112.78 (17)	C7—C6—C5	111.39 (16)
N1—C1—H1A	109.0	C7—C6—H6A	109.4
C2—C1—H1A	109.0	C5—C6—H6A	109.4
N1—C1—H1B	109.0	C7—C6—H6B	109.4
C2—C1—H1B	109.0	C5—C6—H6B	109.4
H1A—C1—H1B	107.8	H6A—C6—H6B	108.0
C1—C2—C3	113.25 (18)	N2—C7—C6	112.51 (15)
C1—C2—H2A	108.9	N2—C7—H7A	109.1
C3—C2—H2A	108.9	C6—C7—H7A	109.1
C1—C2—H2B	108.9	N2—C7—H7B	109.1
C3—C2—H2B	108.9	C6—C7—H7B	109.1
H2A—C2—H2B	107.7	H7A—C7—H7B	107.8
C4—C3—C2	113.58 (17)	C1—N1—H1C	109.5
C4—C3—H3A	108.9	C1—N1—H1D	109.5
C2—C3—H3A	108.9	H1C—N1—H1D	109.5
C4—C3—H3B	108.9	C1—N1—H1E	109.5
C2—C3—H3B	108.9	H1C—N1—H1E	109.5
H3A—C3—H3B	107.7	H1D—N1—H1E	109.5
C5—C4—C3	113.76 (17)	C7—N2—H2C	109.5
C5—C4—H4A	108.8	C7—N2—H2D	109.5
C3—C4—H4A	108.8	H2C—N2—H2D	109.5
C5—C4—H4B	108.8	C7—N2—H2E	109.5
C3—C4—H4B	108.8	H2C—N2—H2E	109.5
H4A—C4—H4B	107.7	H2D—N2—H2E	109.5
C4—C5—C6	114.37 (17)	O2—N3—O3	119.83 (18)
C4—C5—H5A	108.7	O2—N3—O1	121.37 (19)
C6—C5—H5A	108.7	O3—N3—O1	118.72 (18)
C4—C5—H5B	108.7	O5—N4—O6	120.76 (16)
C6—C5—H5B	108.7	O5—N4—O4	120.40 (16)
H5A—C5—H5B	107.6	O6—N4—O4	118.84 (16)
			
N1—C1—C2—C3	173.40 (19)	C3—C4—C5—C6	178.89 (19)
C1—C2—C3—C4	178.6 (2)	C4—C5—C6—C7	172.16 (18)
C2—C3—C4—C5	172.1 (2)	C5—C6—C7—N2	−178.84 (16)

### Hydrogen-bond geometry (Å, °)

**Table:**

*D*—H···*A*	*D*—H	H···*A*	*D*···*A*	*D*—H···*A*
N1—H1C···O4^i^	0.89	2.16	2.956 (2)	149
N1—H1C···O6^ii^	0.89	2.62	3.129 (2)	118
N1—H1D···O1^ii^	0.89	2.09	2.951 (2)	161
N1—H1D···O3^ii^	0.89	2.40	3.035 (2)	129
N1—H1E···O1^i^	0.89	2.04	2.871 (2)	155
N1—H1E···O2^i^	0.89	2.43	3.208 (3)	147
N2—H2C···O2^iii^	0.89	2.26	3.142 (2)	172
N2—H2C···O3^iii^	0.89	2.26	2.911 (2)	130
N2—H2D···O4^iv^	0.89	2.07	2.901 (2)	155
N2—H2E···O4^v^	0.89	2.44	3.0064 (19)	122
N2—H2E···O6^v^	0.89	2.08	2.967 (2)	171

Symmetry codes: (i) *x*+1/2, −*y*+3/2, *z*−1/2; (ii) *x*−1/2, −*y*+3/2, *z*−1/2; (iii) −*x*+3/2, *y*−1/2, −*z*+3/2; (iv) −*x*+1, −*y*+1, −*z*+2; (v) *x*+1, *y*, *z*.
